# Mathematical modeling of therapeutic neural stem cell migration in mouse brain with and without brain tumors

**DOI:** 10.3934/mbe.2022119

**Published:** 2022-01-07

**Authors:** Justin Gomez, Nathanael Holmes, Austin Hansen, Vikram Adhikarla, Margarita Gutova, Russell C. Rockne, Heyrim Cho

**Affiliations:** 1 Department of Mathematics, University of California, Riverside, Riverside, CA 92521, USA; 2 Division of Mathematical Oncology, Department of Computational and Quantitative Medicine, Beckman Research Institute, City of Hope National Medical Center, Duarte, CA 91010, USA; 3 Department of Stem Cell Biology and Regenerative Medicine, Beckman Research Institute, City of Hope National Medical Center, Duarte, CA 91010, USA

**Keywords:** neural stem cell therapy, intranasal drug administration, mathematical oncology, agent based modeling, glioma, LM-NSC008

## Abstract

Neural stem cells (NSCs) offer a potential solution to treating brain tumors. This is because NSCs can circumvent the blood-brain barrier and migrate to areas of damage in the central nervous system, including tumors, stroke, and wound injuries. However, for successful clinical application of NSC treatment, a sufficient number of viable cells must reach the diseased or damaged area(s) in the brain, and evidence suggests that it may be affected by the paths the NSCs take through the brain, as well as the locations of tumors. To study the NSC migration in brain, we develop a mathematical model of therapeutic NSC migration towards brain tumor, that provides a low cost platform to investigate NSC treatment efficacy. Our model is an extension of the model developed in Rockne et al. (PLoS ONE 13, e0199967, 2018) that considers NSC migration in non-tumor bearing naive mouse brain. Here we modify the model in Rockne et al. in three ways: (i) we consider three-dimensional mouse brain geometry, (ii) we add chemotaxis to model the tumor-tropic nature of NSCs into tumor sites, and (iii) we model stochasticity of migration speed and chemosensitivity. The proposed model is used to study migration patterns of NSCs to sites of tumors for different injection strategies, in particular, intranasal and intracerebral delivery. We observe that intracerebral injection results in more NSCs arriving at the tumor site(s), but the relative fraction of NSCs depends on the location of injection relative to the target site(s). On the other hand, intranasal injection results in fewer NSCs at the tumor site, but yields a more even distribution of NSCs within and around the target tumor site(s).

## 1. Introduction

Effective stem cell-based therapies for treatment of brain tumors and repair of damaged brain tissues require efficient delivery of stem cells to the tumor or injury site. One approach exploits the inherent tropism of human neural stem cells (NSCs) to sites of central nervous system (CNS) damage and inflammation for delivery of therapeutics as well as eventual cell replacement and/or stimulation of regeneration [1–3]. NSCs have also been engineered to deliver a variety of anti-cancer agents, and have shown therapeutic efficacy in preclinical models of several types of primary and metastatic brain tumors. These promising results have led to a first-in-human clinical trial of NSC-mediated therapy for glioma patients (clinical trial ID #NCT01172964). In addition, the California Institute for Regenerative Medicine (CIRM) is currently supporting preclinical investigations and clinical trials for development of NSCs for repair of damaged neural tissue associated with stroke, multiple sclerosis and other neurodegenerative diseases. Despite early successes and the promise of these emerging approaches, a major obstacle to further enhancing the efficacy of NSC-based therapy is ensuring that sufficient numbers of viable cells reach the diseased or damaged areas in the CNS. To accomplish this, we and others have explored intravenous (IV), intracranial (IC), and intranasal (IN) administration for delivery of NSCs to the CNS [4, 5]. Although these routes potentially have wide application to CNS tumor therapy, successful translation to the clinic has been hindered by an inability to visualize, quantitatively analyze, and predict migration of exogenous stem cells. To fill this methodological gap, we propose to develop and validate a computational model of NSC migration in the brain based on tissue anisotropy that will allow us to predict NSC migration paths and eventual biodistribution at brain tumor sites.

We, and others, have found that mice bearing orthotopic brain tumors and treated with IC or IN administered therapeutic NSCs show reduced tumor growth and improved long-term survival [4, 6–8]. In addition, the administered therapeutic NSCs specifically localize to brain tumor sites but are not found in non-tumor areas of the brain. However, as with previous studies of IV and IC administered therapeutic stem cells, clinical translation of NSC-based therapy has been hampered by our inability to quantify or predict NSC migration to sites of tumor/injury. This is needed because the paths that NSCs take to the tumors, as well as the location of tumors within the brain, may affect the final number of viable cells that reach the tumor/injury site. We expect that defining how NSCs migrate and how migration paths affect NSC numbers and viability at the tumor/injury site will ultimately allow for disease- or injury site-specific modification of NSC dose and route of administration. We have recently published a manuscript describing biodistribution and tumor coverage of brain tumors by therapeutic NSCs in orthotopic xenograft models of glioma after IC and IV routes NSC administration using 3-dimensional reconstructions [6]. Because the vast majority of studies on IC- or IN-administered therapeutic NSCs to date have been done in rodents, to facilitate the translation from rodents to humans, we propose to first apply and test quantitative anisotropy-based computational methods in already existing preclinical brain tissue sections from brain tumor-bearing mice that received IC- or IN-administered NSCs, and then to validate these methods in preclinical animal studies.

Based on our preliminary data described below, we hypothesize that NSCs migrate along white matter tracts in the brain, and that the resulting routes of migration are predictable and depend on the spatial relationships of sites of NSC administration, tumor targets, and intervening white matter tracts. Technologies currently exist to map white matter tracts and fluid gradients in the living brain (including human brain), such as diffusion tensor imaging (DTI) [9]. Diffusion tensor tractography is a computational method of connecting regions in the brain that is based on the anisotropy, or directed orientation of the tissue. Myelinated axons in the white matter of the brain are very spatially oriented, or highly anisotropic, whereas the grey matter that composes the brain cortex is dense and lacks distinct spatial orientation. DTI and tractography are established in humans and are routinely collected clinical images in neuro-radiology and can be used to model migration of cells in the brain [10, 11]. Moreover, various cytokines and chemokines are involved in NSC migration which makes the prediction more challenging. In particular, urokinase plasminogen activator (uPA) and urokinase plasminogen activator receptor (uPAR) are shown to be involved in the migration of NSCs to malignant tumors, as well as various cytokines including interleukin-6 (IL-6), interleukin-8 (IL-8), and monocyte chemoattractant protein-1 [12–14]. Thus, the proposed studies using mechanistic modeling will provide an important preclinical model system and, because the methods can be established in humans, it will be feasible to translate results from pre-clinical studies to clinical trials in humans.

In silico models have long been established as a cost-effective and efficient computational alternative to in vivo and in vitro experiments. Various modeling approaches have been developed to understand the complex mechanisms of tumor growth and treatment responses [15, 16]. These include discrete approaches such as cellular automata and agent-based modeling [17–19]. Continuum approaches are a good alternative for tissue scale simulations, using ordinary differential equations [20–23], partial differential equations [24–26], and integro-differential equations [27–29]. Multi-scale models that combine representations of the tumor microenvironment at subcellular, cellular, and tissue scales have been developed as well [30–32]. See reviews [33–37] for different mathematical modeling approaches. Such models provide quantitative tools for testing therapies that improve treatment response and circumvent unfortunate consequences such as transient regression or relapse.

Mathematical models of cell migration and tumor invasion has been developed using a range of models in recent years. Cancer cells must exhibit invasive behavior through the surrounding tissue for metastasis to occur. Thus, many mathematical models have been developed to understand the mechanisms that allows cancer to invade through the components of the extracellular matrix by interacting with the microenvironment and surrounding cells [38, 39]. The effects of various components in the invasive caner system have been studied, including vascularization, matrix-degradative enzyme such as matrix metalloproteinases (MMPs), protease such as urokinase plasminogen activator (uPA), cell-cell adhesion, and cell-matrix adhesion [40–43]. See the recent review in [44]. Many models have been specifically developed for brain cancer [45–47]. While earlier models assumed isotropic and homogeneous migration, it became clear that the complex tissue structure should be taken into account to model the aniotropic nature of glioma invasion. [11] models the directed movement of cells along the aligned neural fiber tracts of white matter using DTI data. This work shows that information of the direction and degree of anisotropy obtained from the diffusion tensors is effective in modeling the anisotropic structure of brain tissue. [48] extends the aforementioned model by including adhesion mechanisms between the glioma cells and the extracellular matrix associated to white matter tracts, and demonstrates that the adhesion mechanism is crucial to explain thin invasive front of glioma. Patient-specific model of brain tumor has been studied in [49] by calibrating the DTI derived glioma invasion model to data of 10 patients. This work compares the prediction between isotropic and anisotropic model and investigates the level of anisotropy that anisotropic model makes better prediction compared to isotopic model. While there are extensive list of literature on modeling cancer cell migration, including glioma invasion, there are less number of work on modeling the NSC migration. One of the few models is an agent based model developed in Rockne et al. (PLoS ONE 13(8), e0199967, 2018) [10], that models the migration of NSC as directed movement along the white matter tract using DTI data.

In this paper, we develop a mathematical model that describes the delivery of therapeutic NSCs in mouse brain with glioma. The remainder of this paper is structured as follows. The NSC migration model in normal brain without cancer is summarized in Section 2.1. In Sections 2.2 and 2.3, we extend the model developed in Rockne et al. (PLoS ONE 13(8), e0199967, 2018) [10] by adding chemotaxis and stochasticity within the population. While the former model only included directed migration of NSCs along white matter tracts, our model includes additional directed migration due to uPA concentration via chemotaxis. In addition, we model stochasticity within the population in their migration speed and sensitivity to chemotaxis. The effects of these added parameters are studied in Section 3.1, where we examine the distance of cell migration, proportion on white matter tracts, and cancer arrival rate. In Section 3.2, intranasal injection and intracerebral injection strategies are compared for three different scenarios regarding the location of glioma. In particular we study tumor sites relatively close to and farther away from the white matter tract, as well as two tumor sites on the opposite side of the brain. Finally, a summary of our work and future directions are discussed in Section 4.

## 2. NSC migration model

**Figure 1.**
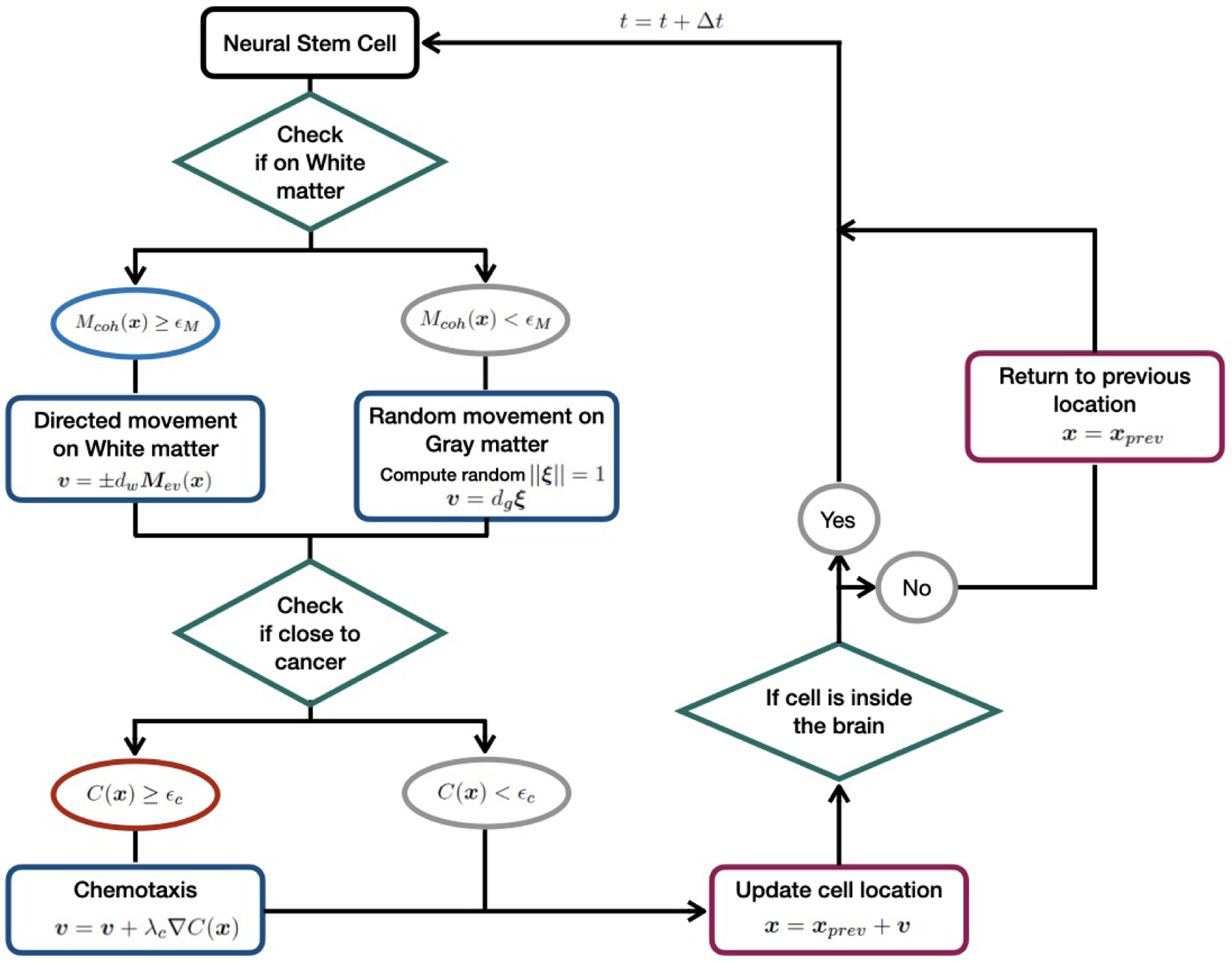
Summary diagram of the NSC migration model with chemotaxis.

The mathematical model of NSC migration in the 3-dimensional mouse brain is presented in this section. Each subsection describes the parts of our model that extends [10], namely, three-dimensional migration, chemotaxis, and stochasticity. The overall diagram of migration model for individual NSC is shown in Figure 1.

### 2.1. NSC migration model along white matter tracts

NSCs are known to migrate along the white matter tracts in the brain. In particular, the correlation between the orientation of NSC and anisotropy of white matter has been recognized in [10]. Accordingly, the direction of migration of NSCs is determined by the structural orientation of the brain tissue, computed with structure tensor analysis. The eigenvectors and corresponding eigenvalues of the structure tensor are used to compute the direction and relative orientation of both the white and grey matter composing the brain tissue. For brain image data ***I***(***x***), the structure tensor at ***x*** is defined as following.


$$T_{\sigma }(x)=\left[\begin{array}{cc} I_{x}{\left(x\right)}^{2} & I_{x}(x)I_{y}(x)\\ I_{x}(x)I_{y}(x) & I_{y}{\left(x\right)}^{2} \end{array}.\right]$$

The eigenvectors and eigenvalues of the structure tensor are denoted ***e***_*i*_ and *λ*_*i*_, respectively. In [10], a model for NSC migration in two-dimensions has been developed, where the anisotropy of white matter is quantified using the coherence of the structure tensor, as

$$M_{coh}^{2D}={\left(\frac{\lambda_{1}-\lambda_{2}}{\lambda_{1}+\lambda_{2}}\right)}^{2}.$$

Here, *λ*_1_ and *λ*_2_ are the eigenvalues of diffusion tensor which can be ordered so that *λ*_1_ ≥ *λ*_2_ > 0 and at least one eigenvalue is assumed to be nonzero. The eigenvector that corresponds to the largest eigenvalue, ***e***_1_ = (*e*_*x*_, *e*_*y*_) is converted into its angle as *M*_*ang*_ = tan^−1^(*e*_*y*_/*e*_*x*_) if *e*_*x*_ ≠ 0, and *M*_*ang*_ = π if *e*_*x*_ = 0. In three-dimensions, fractional anisotropy can be used to describe the degree of anisotropy. Fractional anisotropy is calculated as

$$M_{coh}^{3D}=\sqrt{\frac{3}{2}}\frac{\sqrt{{\left(\lambda_{1}-\hat{\lambda }\right)}^{2}+{\left(\lambda_{2}-\hat{\lambda }\right)}^{2}+{\left(\lambda_{3}-\hat{\lambda }\right)}^{2}}}{\sqrt{\lambda_{1}^{2}+\lambda_{2}^{2}+\lambda_{3}^{2}}},$$

where *λ*_1_, *λ*_2_, and *λ*_3_ are the eigenvalues of diffusion tensor, and $\hat{\lambda }=\left(\lambda_{1}+\lambda_{2}+\lambda_{3}\right)/3$ is the mean of the eigenvalues.

The governing equation of the NSC migration in non-tumor bearing naive brain is as follows. In both two- and three-dimensional models, a region is regarded as white matter if *M*_*coh*_ is larger than a certain threshold value, denoted as *ϵ*_*M*_. Thus, if

$$M_{coh}(x)\geq \epsilon_{M},$$

***x*** is regarded as white matter, and otherwise grey matter. The cells in white matter will follow the elongated direction of white matter tract, that is given by the eigenvector ***e***_1_ that corresponds to the largest eigenvalue. Otherwise, if the cells are in grey matter, they will not have any preferred direction, and we model NSC movement as a random walk.

(2.1) $$v=\{\begin{array}{ll} \pm d_{w}M_{ev}(x), & M_{coh}(x)\geq \epsilon_{M}\\ d_{g}\xi (\omega ), & M_{coh}(x)<\epsilon_{M} \end{array}$$

where ***M***_*ev*_(***x***) = ***e***_1_(***x***)/∥***e***_1_(***x***)∥ is the normalized direction of white matter, ***ξ***(*ω*) is a random sample of unit length, and *d*_*w*_ and *d*_*g*_ are step sizes. The random walk ***ξ*** can be computed as follows. In the two-dimensional model, $\xi_{i}(\omega )\in {\mathbb{R}}^{2}$ is a random sample on a unit circle boundary, and $\xi_{i}(\omega )\in {\mathbb{R}}^{3}$ is a random sample on a unit sphere surface in the three-dimensional model, such that, ∥***ξ***_*i*_∥ = 1. Among the two directions, ***M***_*ev*_ or −***M***_*ev*_, that NSC can take, we assume that NSCs choose the direction that is consistent with the previous step, assuming that the movement of NSCs are under the influence of inertia. To compute such direction, at time step *t*_*i*_, we take the sign of the inner product between ***x***_*i*_−***x***_*i*−1_ and ***M***_*ev*_, that is,

$$v=\left[\text{sign}\left(\left(x_{i}-x_{i-1}\right)\cdot M_{ev}\left(x_{i}\right)\right)\right]d_{w}M_{ev}(x).$$

Although in our current model, the NSCs strictly follow the white matter tract once it is on it, a noise term could be added to allow stochastic variation, e.g., *v* = ±*d*_*w*_ ***M***_*ev*_(*x*) + *ϵ*. We will describe in the following section that NSCs can leave the white matter tract via chemotaxis signal.

### 2.2. NSC migration model with chemotaxis

In addition to the migration along the white matter tract, we model the tumor-tropic migration toward cancer by chemotaxis. In particular, we consider NSCs to be sensitive to uPA concentration that is known to be significantly higher in glioma than in normal brain tissue [12]. Moreover, hypoxia and metastasis is known to induce overexpression of the uPA and its receptor. To model the chemotaxis movement of NSCs, we consider the concentration of uPA as *C*(*x*, *y*, *z*). We assume that the time scale of uPA diffusion is significantly longer than the time scale of the NSC migration, thus consider *C*(*x*, *y*, *z*) to be time-independent. uPA concentration is modeled to decay from the center of the tumor (*x*_*c*_, *y*_*c*_, *z*_*c*_), as

$$C(x,y,z)={\left(1+\sqrt{\frac{{\left(x-x_{c}\right)}^{2}}{\sigma_{x}^{2}}+\frac{{\left(y-y_{c}\right)}^{2}}{\sigma_{y}^{2}}+\frac{{\left(z-z_{c}\right)}^{2}}{\sigma_{z}^{2}}}\right)}^{-p},$$

where (*σ*_*x*_, *σ*_*y*_, *σ*_*z*_) represents the distance from the tumor that the substance is halved in each *x*, *y*, and *z* direction, and *p* represents how gradually the substance decay. Chemotaxis is the movement of cells in a direction corresponding to a chemical stimulus, for example, an increasing uPA concentration in our case. Therefore we include the gradient of uPA concentration *λ*_*c*_∇*C*(***x***) = *λ*_*c*_(*∂*_*x*_*C*, *∂*_*y*_*C*, *∂*_*z*_*C*) to Eq (2.1) if uPA concentration *C*(***x***) is above a certain threshold, denoted as *ϵ*_*c*_. Here, *λ*_*c*_ is the chemotaxis sensitivity parameter.


(2.2) $$v=v+\lambda_{c}\nabla C(X(t)),\;C(x)\geq \epsilon_{c}.$$

### 2.3. Stochasticity within the population

NSCs have demonstrated stochasticity in their migration distance and response to chemotactic signals. While some NSCs are able to quickly migrate to the intended tumor site, many of them are found near the injected location and as well as other parts of the brain. Therefore, we choose the migration speed and chemotaxis sensitivity as stochastic parameters.

#### 2.3.1. Stochastic migration speed

Stochasticity of NSC migration speed is modeled with a beta distribution, B[*α*, *β*]. Instead of the deterministic migration speed *d*_*w*_ in Eq (2.1), we consider a stochastic migration speed

$$d_{w}\psi (\omega ),\;\psi (\omega )\sim \text{B}\left[1,\beta_{w}\right],$$

where *β*_*w*_ ≥ 1. Note that we take *α* = 1 so that *β*_*w*_ = 1 will result in a uniform distribution, and we consider *β*_*w*_ ≥ 1 so that more cells are likely to have small speed. This choice is due to the experimental results showing that some NSCs move relatively fast, but the majority of cells do not move much from the injection site.

#### 2.3.2. Stochastic chemo-sensitivity

Chemosensitivity of NSCs are also assumed to be stochastic. We again consider a beta distribution instead of the deterministic chemosensitivity parameter *λ*_*c*_ in Eq (2.2), we consider

$$\lambda_{c}\eta (\omega ),\;\eta (\omega )\sim \text{B}\left[\alpha_{c},1\right].$$

Similarly, *α*_*c*_ = 1 will result in a uniform distribution of chemosensitivity from 0 to *λ*_*c*_ among the NSCs, and as *α*_*c*_ increases as *α*_*c*_ ≫ 1, the chemosensitivity will be closer to being deterministic.

## 3. Simulation

In this section, the migration patterns of NSCs in the mouse brain are studied using our model. We consider LM-NSC008 cells [4] with the range of parameter values chosen similar to [10] as shown in Table 1. In addition, we include functions and parameters to model chemotaxis and stochasticity, which we study the effect of in Section 3.1. In Section 3.2, we further study the model in the three-dimensional mouse brain, where we focus on comparing two different injection strategies, intracerebral and intranasal routes of delivery. We initialize our simulation with 1,000 NSCs. Time step is chosen as Δ*t* = 1/1,000 days. In both the 2-dimensional and 3-dimensional models, we begin by initializing our NSC by randomly generating them within a given radius of the injection site. Then each NSC at every Δ*t* performs one of two actions: if it is on the white matter, it migrates along the direction of the white matter tract, or if it is on the grey matter, it moves to a random direction. In addition, the migration step due to chemotaxis is appended. We repeat this process up to 30 days.

### 3.1. Parameter study in 2D

First, we study how variations in our model parameters effect NSC migration and chemotaxis. We use a two-dimensional cross-section of mouse brain and intracerebral delivery using mouse DTI from [10]. The image is a vertical cross-section of a mouse brain with size 8,892× 5,004 pixels and length 1.444 *μm* per pixel. For this study, we consider two tumor locations, one close to the injection site in the frontal lobe (Tumor 1) and another further away, past the anterior commissure (Tumor 2). The tumor locations are plotted in Figure 4 in red circles.

#### 3.1.1. NSC migration speed

**Figure 2.**
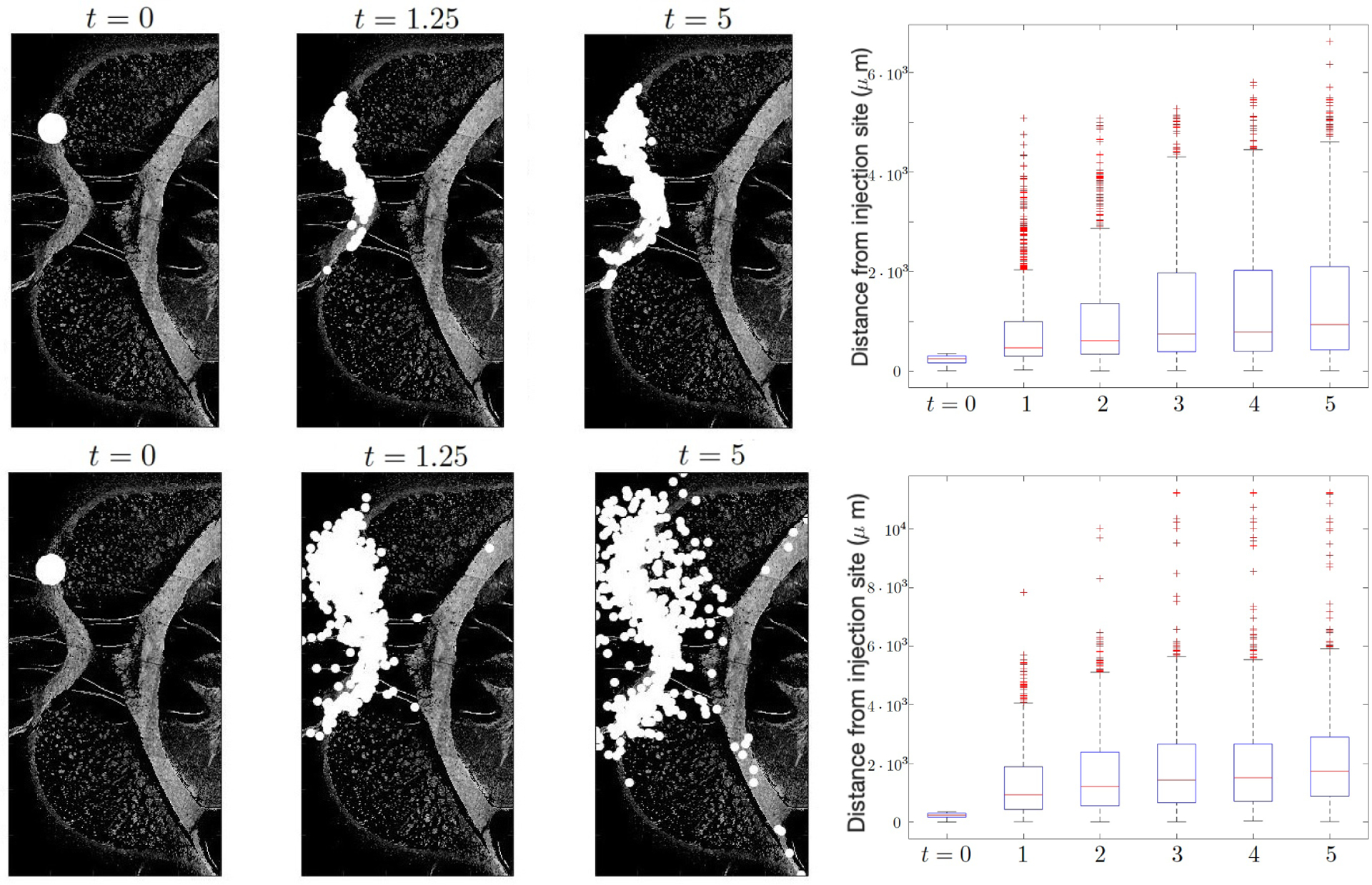
NSC distribution comparing different values of grey matter migration speed, *d*_*g*_, using *d*_*w*_ = 5, *d*_*g*_ = 0.5 (top) and *d*_*w*_ = *d*_*g*_ = 5 (bottom). Note that using *d*_*w*_ = *d*_*g*_ results in scattered NSC with many of them located outside the white matter. When NSCs migrate slower in gray matter, such as *d*_*g*_ = 0.5, they stay mostly in the white matter. Furthermore, NSCs only arrive at the anterior commesure when the gray matter migration speed is large enough, e.g., *d*_*g*_ = 5. The boxplot shows the distance NSCs traveled from injection site, where we observe the NSC migration distance approximately doubles when *d*_*g*_ = 5 compared to *d*_*g*_ = 1.

The maximum migration speed of NSCs in white matter is parameterized by *d*_*w*_ and in gray matter by *d*_*g*_. We fix *d*_*w*_ = 5 so that the migration speed is *d*_*w*_× 1.444 *μm* per time step, Δ*t* = 1/1,000 in days. Figure 2 compares the migration patterns using different values of *d*_*w*_ and *d*_*g*_. When the migration speed is slower in the gray matter, such as *d*_*g*_ = 0.5 and *d*_*w*_ = 5, the NSCs mostly stay on the white matter tracts. On the other hand, when the migration speeds are identical, such as *d*_*w*_ = *d*_*g*_ = 5, NSCs are more randomly distributed across the entire brain. We also observe that cells arrive at the anterior commissure only when *d*_*g*_ is large enough, for example when *d*_*g*_ = 5. Among the 1,000 cells that are injected, cell numbers in the order of O(10) reach the anterior commissure at *t* = 3.75 days following NSC injection. Further, we quantify the distance from the injection site. The box-plot in Figure 2 compares the distance traveled by NSCs for different values of *d*_*g*_. The median of distance from the injection site on day 5 increases from around 1,000 *μm* when *d*_*g*_ = 0.5 to 2,000 *μm* when *d*_*g*_ = 5.

**Figure 3.**
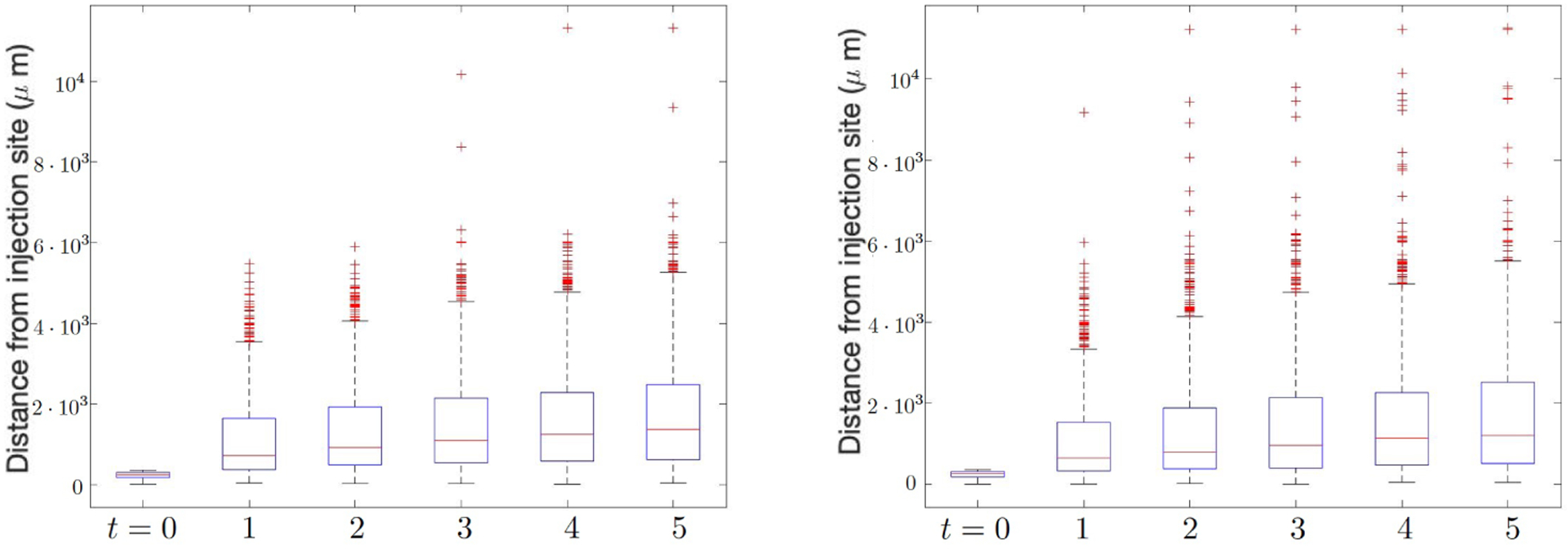
Distance NSCs traveled from injection site comparing different values of stochastic migration speed, *β*_*w*_ = 1 (left) and *β*_*w*_ = 4 (right). We observe an increasing number of NSC outliers both sooner and in total as *β*_*w*_ increases, while the median distance decreases.

We modeled the maximum migration speed *d*_*w*_ to be stochastic, in particular, we considered a beta distribution, *B*[1, *β*_*d*_] for *d*_*w*_. The first parameter is fixed at 1 so that the distribution is skewed toward zero. This is done to reflect experimental results, which show that fewer cells travel rapidly, while most cells stay close to the injection site. To study the effect of this, we vary the second parameter *β*_*w*_ ∈ [1, 4]. When *β*_*d*_ = 1, the migration speed of the population is uniformly distributed in [0, *d*_*w*_], however, as *β*_*w*_ increases, the distribution will be skewed more towards zero. In addition, we rescale the values so that the average is consistent with the deterministic case, so that there will be more outliers that have large migration speed as we increase *β*_*w*_. The results of comparing *β*_*w*_ = 1 and *β*_*w*_ = 4 are shown in Figure 3, where an increased value of *β*_*w*_ yields more NSCs that travel above a distance of 8,000 *μm*, while the median distance decreases. We comment that the effect of larger migration speed *d*_*g*_ remains when stochasticity is added. For fixed *β*_*w*_ = 1, increasing *d*_*g*_ results in more NSCs migrating a longer distance arriving at the anterior commissure. Increasing *β*_*w*_ with fixed *d*_*g*_ and *d*_*w*_ gives the same result.

#### 3.1.2. Chemotaxis and sensitivity to chemoattractant

**Figure 4.**
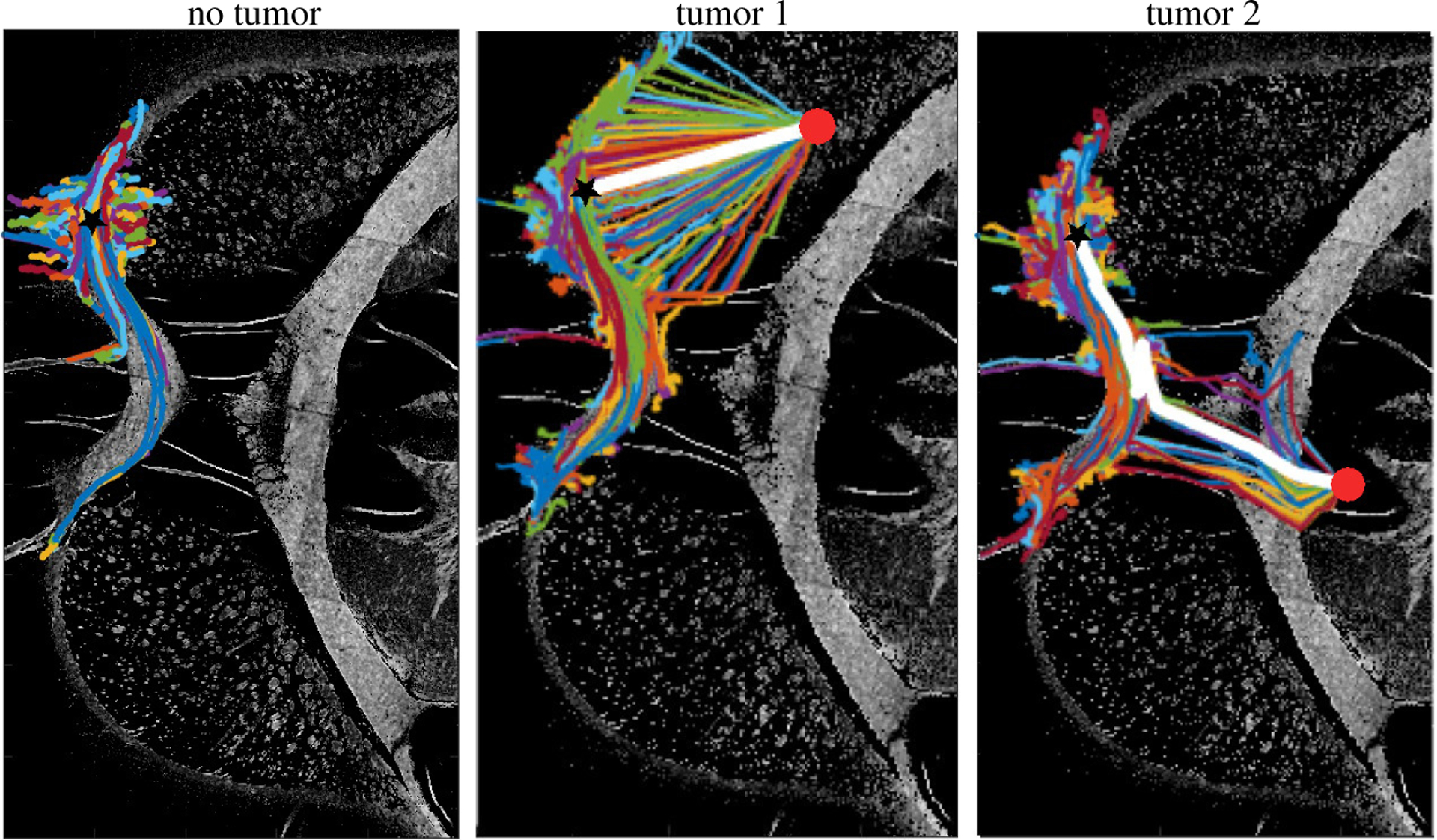
Cell trajectories of NSC migration without cancer (left) and with cancer (middle, right). The injection site and cancer is marked with a black star and a red circle, respectively. In the normal case, NSCs migrate along white matter tracts, however, when cancer is present, the cells robustly migrate to the cancer site when the cancer is in the frontal lobe, close to the injection site. In case the tumor further away past the anterior commissure, NSCs travel first along the white matter tract before they get close enough to the cancer site and pick up the chemotaxis signal. The minimal migration path is marked in white line.

Figure 4 compares the trajectory of NSC migration in the case of non-tumor bearing naive brain as compared to when a glioma brain tumor is present. Without a tumor to target, the NSCs migrate along the white matter tracts in the corpus callosum, and mostly stay on the tracts. However, when a tumor is present in the middle of corpus callosum and anterior commissure, NSCs effectively migrate to the cancer site by the chemotaxis mechanism. Note that we use the chemotaxis sensitivity parameter value as *λ*_*c*_ = 6.

In addition, we compare two tumor sites as shown in Figure 4. The first tumor (Tumor 1) is located in the right frontal lobe between the corpus callosum and the anterior commissure which is closer to the injection site. The other tumor (Tumor 2) is located further away from the injection site past the anterior commissure. The white line represents the shortest migration path of NSC from the injection to the cancer site. We observe distinct migration patterns in the two tumor sites. When the cancer is located closer to the injection site, NSCs can directly migrate to the cancer site. However, when the cancer is located further away past the anterior commissure, NSCs cannot detect the chemotaxis gradient when they are injected. However, as they migrate along the white matter tract, and the ones that move closer to the cancer site detect the chemoattractant and start moving toward the tumor site.

**Figure 5.**
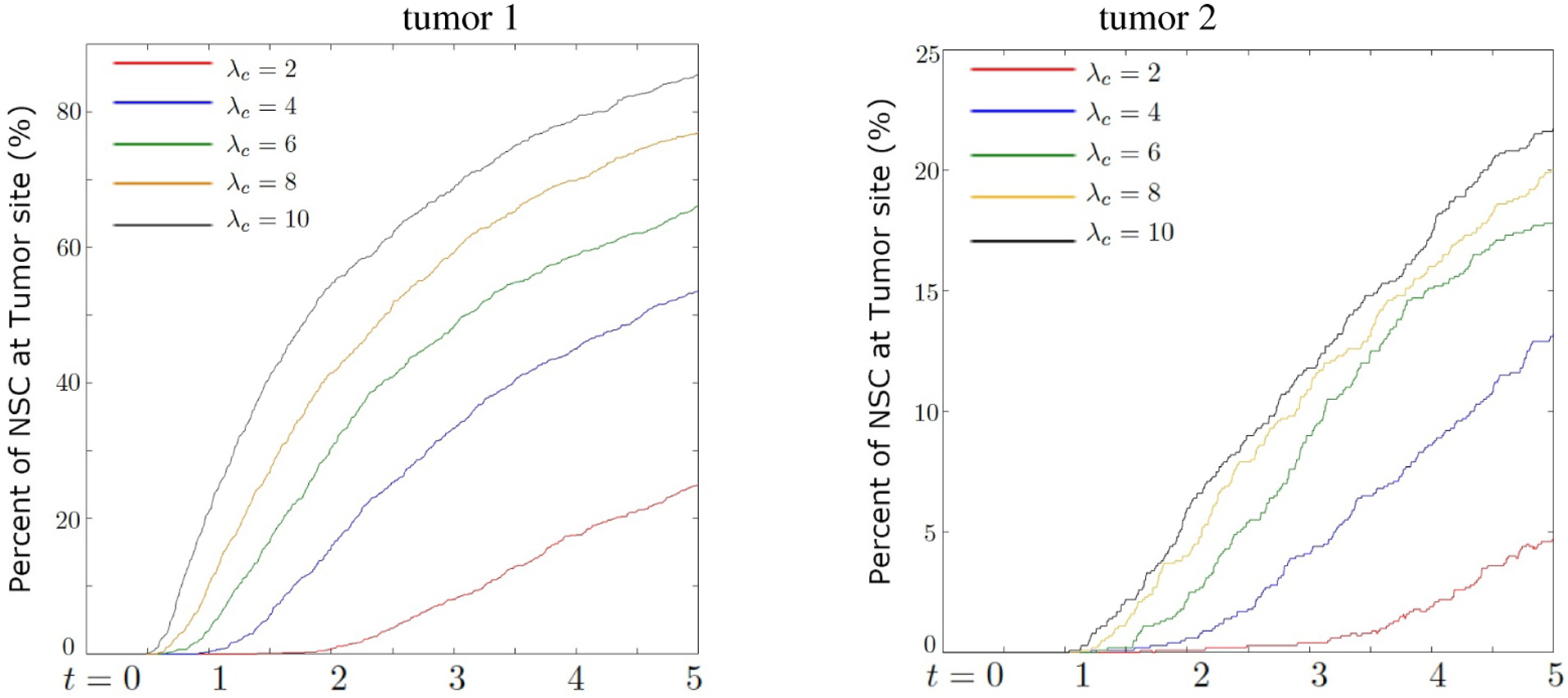
Percentage of NSCs arrived at the tumor site comparing different target locations, frontal lobe (Tumor 1) and further away pass anterior commissure (Tumor 2). As chemosensitivity *λ*_*c*_ increases, the amount of NSC that reaches the cancer site also increases.

Figure 5 shows the percentage of NSCs that arrive at the cancer site for different values of chemosensitivity parameter *λ*_*c*_. As expected, approximately four times more NSCs arrive at the closer site (Tumor 1) as compared to the more distant site (Tumor 2). We also observe that increasing the chemosensitivity parameter *λ*_*c*_ substantially increases the proportion of NSCs that arrive at the cancer. When *λ*_*c*_ = 10 almost 85% arrive at cancer site 1 and above 20% arrive at cancer site 2, despite being further away. We comment that by adding stochasticity to the chemosensitivity parameter, the arrival at the cancer site reduces significantly especially for smaller values of *α*_*c*_. As the stochastic parameter increases as *α*_*c*_ ≥ 4, the results approach the deterministic model with the same chemosensitivity value.

### 3.2. Comparison of intranasal and intracerebral injection in 3D

In this section, we simulate the model on a three-dimensional mouse brain, and focus on comparing two different injection strategies, intracerebral and intranasal injection. Intracerebral administration of NSCs injects cells directly into the brain, which is one of the most direct methods of drug delivery to the target site since it bypasses the blood-brain barrier and other mechanisms that limit drug distribution. However, this method is invasive such that it requires opening the skull, and also the wound from the injection needle can cause a hostile environment for the NSCs to survive. An alternative method is intranasal administration that insufflates the drug through the nose. The therapeutic agents are then transported through the nasal cavity to the olfactory epithelium that covers the upper part, before moving to the olfactory bulb which provides a direct connection between the brain and its external environment. The advantage of intranasal injection over intracerebral is it’s non-invasive nature of administration while similarly bypassing the blood-brain barrier to deliver the drug agents. Also, the possibility of repeated treatment is another major advantage of intranasal administration over intracerebral administration, whereas intracerebral administration can be given only once.

In Table 2, we summarize experimental results of NSC administration from [6] and [55]. In [6], HB1.F3.CD21 NSCs are administered via intracerebral/ventricular route in glioma xenograft mice. For GL261 and PBT017 tumor bearing mice, 4 × 10^5^ NSCs were administered, and the brains were harvested 2–3 days after NSC administration, respectively. The NSCs at cancer sites were quantified by estimating the number of NSC clusters. As presented in Table 2, 773 and 1870 clusters were identified in GL261 cell line and 2076 clusters in PBT017 cell line. Although we cannot calculate the percentage of the arrival exactly, assuming that there are 20 cells per cluster, we estimate 6.61±3.88% and 10.38% of arrival percentage for GL261 and PBT017 cell lines, respectively. On the other hand, intranasal administration is tested in [55]. For U251 glioma bearing mice, 6 × 10^5^ NSCs were administered intranasally. In this experiment, the number of NSCs at cancer site is estimated as 3,000–7,000 cells. This corresponds to arrival percentage of 0.55 ± 0.16% on day 1 and 1.25% on day 4. Although the two experiments are not controlled to be directly comparable, they provide an idea about the efficacy of intracerebral and intranasal administration of NSCs. In addition, we comment that our simulation results of percentage of cancer arrival are overestimated due to not considering the fraction NSCs dying at the injection site. While we assume that all NSCs survive and migrate inside the brain, in reality, only 10–20% of NSC are known to survive after injection.

**Figure 6.**
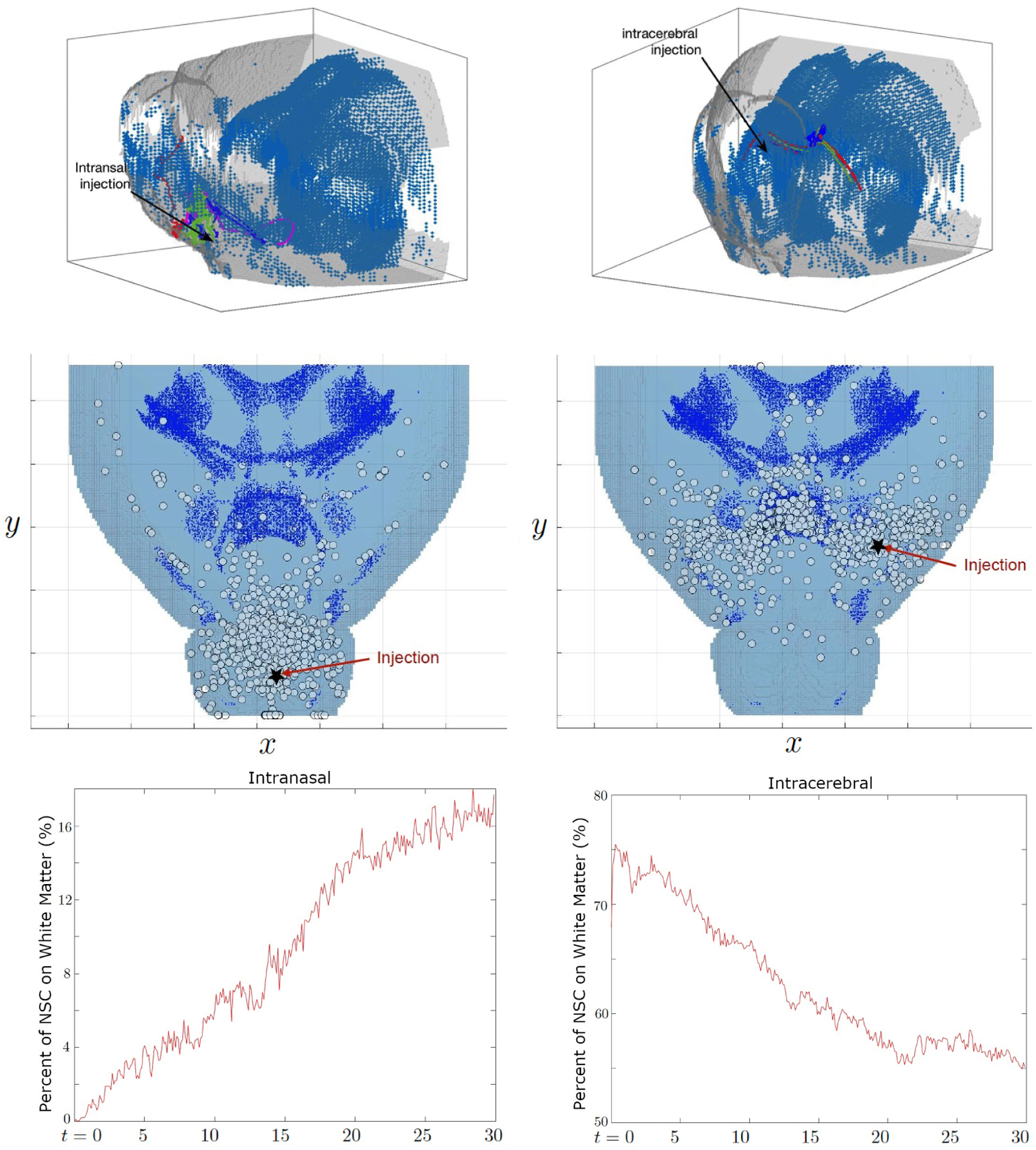
Comparison of intranasal (left) and intracerebral (right) administration of NSCs without the presence of cancer. The white matter tract is marked by blue dots. Selected few trajectories of NSCs (top), the location of NSCs on day 30 (middle), and the percentage of NSCs on white matter tracts (bottom) are shown. NSCs injected by intranasal administration slowly migrate to the center of the brain following the white matter tract in the lower part. On the other hand, NSCs injected in the cerebrum migrate along the white matter tract of the corpus callosum and spread out more easily across the brain. The percentage of intranasal NSCs on white matter tracts trends slowly up to over 16%. In case of intracerebral NSCs, the percentage decays from 70 to 55%, since the cells are injected near the white matter tract, but spread throughout the brain.

We take the mouse DTI from [56] with size 637 × 557 × 277 pixels and 13.5 *μm* per pixel. Figure 6 shows the structure of the mouse brain, where the white matter tract is marked with blue dots. We simulate migration of 1,000 NSCs in both injection strategies, intranasal and intracerebral injection. The injection sites are shown with a few migration paths and distributions of NSCs on day 30. In this simulation, no cancer was present. We observe that NSCs injected through the intranasal route take the white matter tract in the lower part of the brain, but a fraction of the cells remain near the injection site. On the other hand, NSCs administered through intracerebral path migrate along the white matter tract in the corpus callosum. The percentage of NSCs on the white matter tract is shown in Figure 6. In the case of intranasal injection, the percentage gradually increases up to 15% at three weeks whereas for intracerebral, the cells are injected near or on the white matter tract so that the percentage starts higher and decays to around 55 %.

Let us study the two injection methods for brain with glioma. The size of the tumor is chosen to be 200 × 200 × 800 *μm*. We consider the following three scenarios with different locations and numbers of cancer as follows:

- Case 1: One cancer site on the front side of right putamen
- Case 2: One cancer site on the rear side of right putamen
- Case 3: Two cancer sites on the left and right putamen

**Figure 7.**
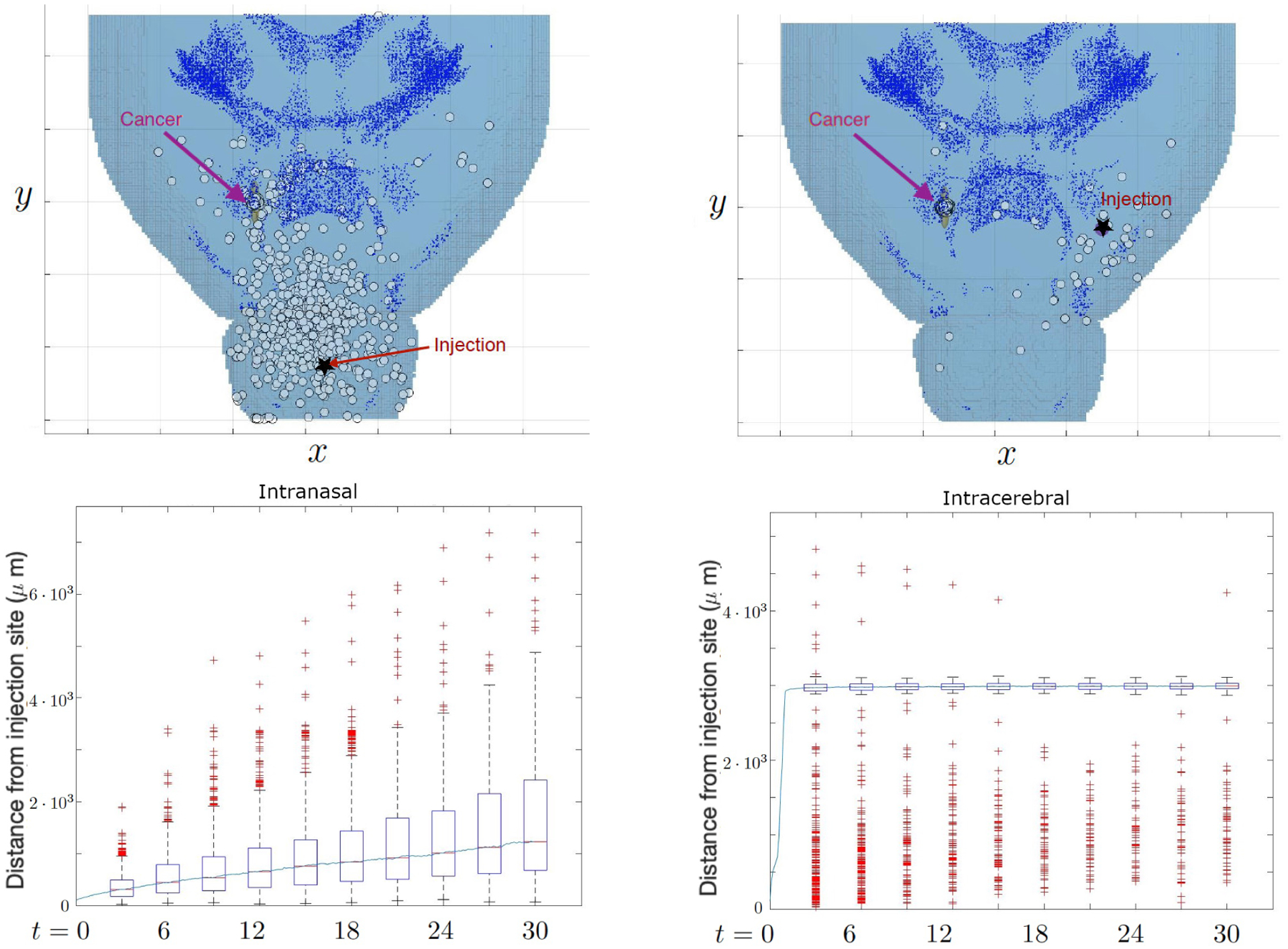
Case 1: Location of NSCs on day 30 (top) and distance NSCs travel from injection site (bottom) starting from intranasal injection site (left) and the intracerebral injection site (right). The NSCs from the intranasal injection spread evenly throughout the simulation. On the other hand, majority of the NSC from the intracerebral injection rapidly migrates towards the cancer site while leaving some outliers near the injection site.

**Figure 8.**
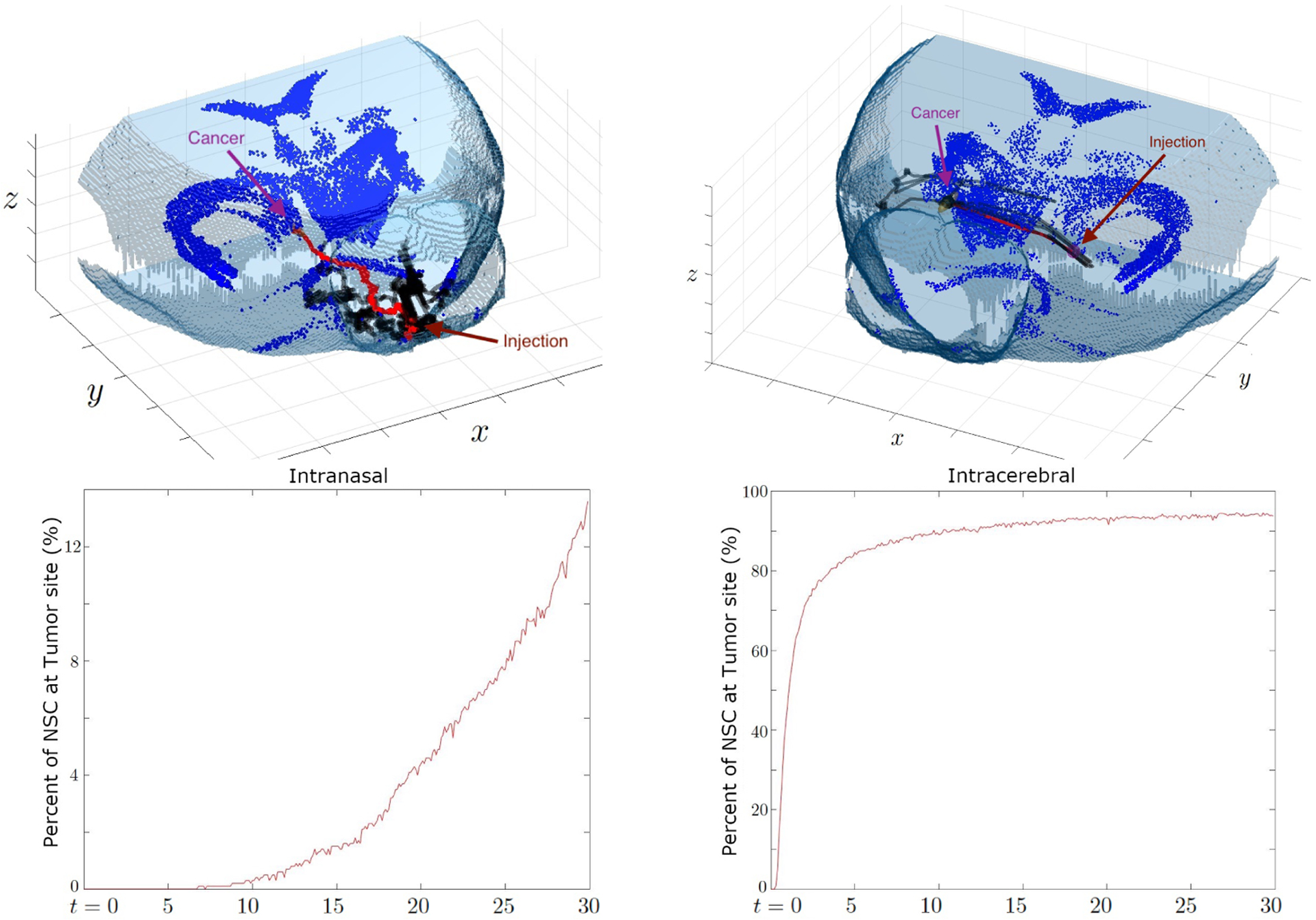
Case 1: The migration paths of selected NSCs towards the tumor centered at the front side of right putamen (top) and the percentage of NSCs that reach the cancer site (bottom). The cells are injected either intranasally (left) and intracerebrally (right). The red line represents the NSC that traveled the shortest path from its initial position to the cancer site. The shortest path taken by the intracerebrally injected NSCs directly follows the major white matter tract, while intranasally injected NSCs have to navigate through a longer distance. NSCs from the intranasal injection site travel gradually to the cancer site, with about 14% reaching their destination on day 30. Meanwhile, NSCs from the intracerebral injection site swiftly travel to the location of the tumor and a much greater percent arrive at around 90%.

We begin our simulations with 1,000 NSCs for the case of cancer site centered at the front side of the right putamen, ***x***_*c*_ = [230, 300, 175]. The movement of NSCs throughout the brain is observed using both methods of injection, intranasal and intracerebral. With this framework for study set in place, Figure 7 shows the distribution of NSCs at the final simulation time, day 30, and some trajectories. We can observe that the NSCs arrived at the cancer location in both injections. In particular, most of the intracerebrally injected NSCs are shown to be at the cancer site. The boxplot showing the distance from the injection site reveals that intracerebral NSCs mostly seem to travel and stay around 3,000 *μm* from the injection site within three days, which agrees with the distance to the cancer site. On the other hand, the intranasal NSCs seem to spread out gradually from their initial starting point, with some outliers most likely indicating those few cells that travel much farther and manage to reach the white matter deeper in the brain. This contrast is due to the cancer site being directly connected to the intracerebral injection site via a white matter tract which functions as the shortest path that the NSCs traverse. In Figure 8, we see that the intracerebral cells are able to take a much more direct path to the cancer location as opposed to the intranasal cells that follow a winding path from the olfactory bulb to the center of the brain. The fraction of NSCs that reached the cancer site, which exemplifies the contrast provided by the difference in starting position. Around 14% of NSCs injected intranasally reach the cancer site, as opposed to the approximately 90% of NSCs injected intracerebrally.

**Figure 9.**
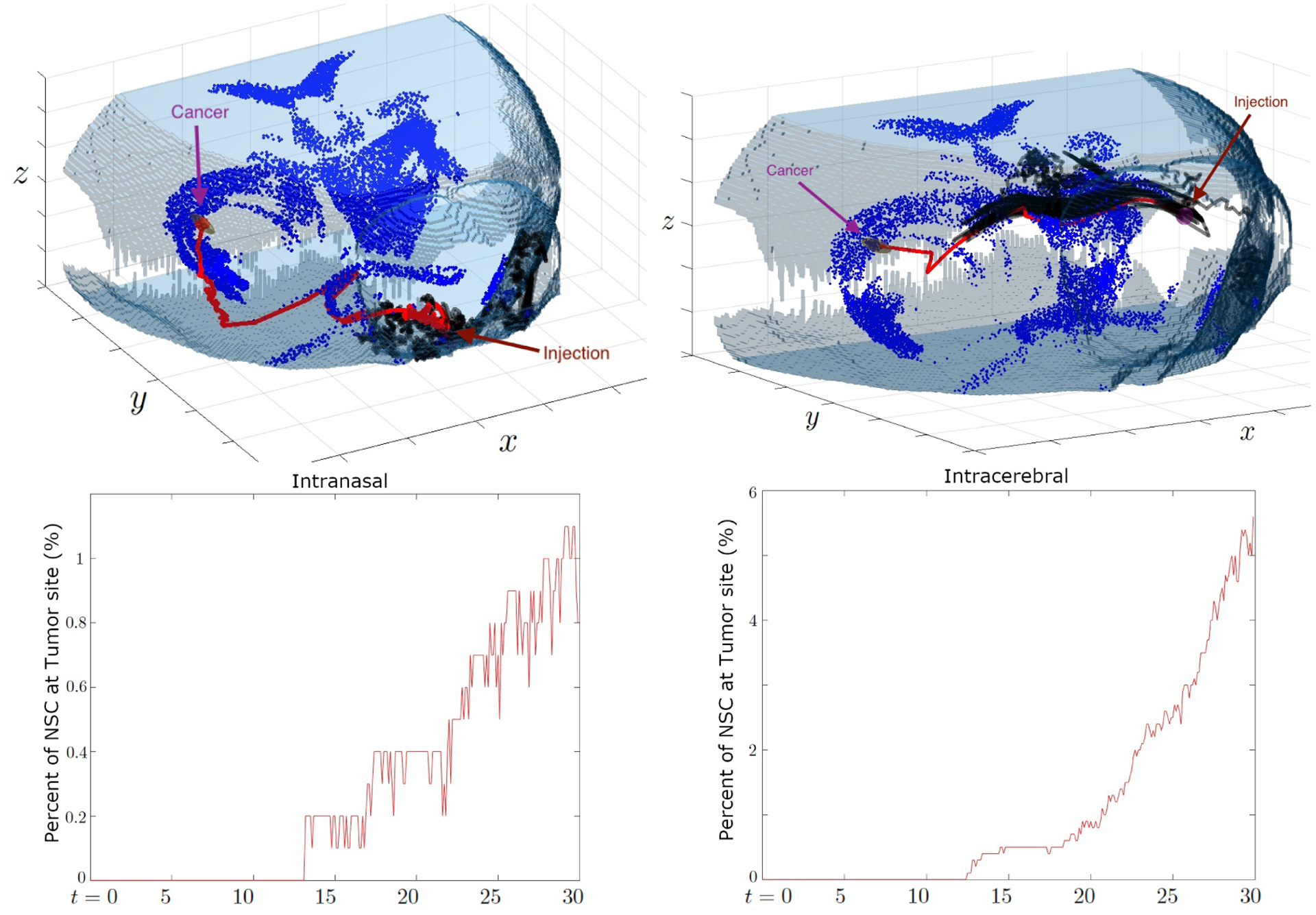
Case 2: The migration paths of selected NSCs towards the tumor centered at the rear side of the right putamen (top) and the percentage of NSCs that reach the cancer site (bottom). The cells are injected either intranasally (left) and intracerebrally (right). We observe that when injected intranasally, only about 1% ever arrive on day 30 while when injected intracerebrally, this number jumps up to 5% total.

The second simulation with cancer present places the cancer site centered at the rear side of the right putamen, ***x***_*c*_ = [120, 400, 150]. Compared to the first simulation, this cancer location is further away from both injection sites making NSC treatment more challenging. Figure 9 shows that the NSC that takes the shortest path from the intranasal injection site to the cancer location navigates through the brain along the white matter tract until it becomes close enough to the cancer and can read the chemotaxis signal. Such a path may not be a straight line to its destination, resulting in it taking a longer route around the brain. This is in contrast with case 1 where the cancer site was relatively close to the intranasal injection site, and both the injection site and cancer were located near a white matter tract causing the shortest path to be more direct. We see that as a result of this challenging tumor location, in both injection strategies, the percentages are fairly low, capping out at around 5% in the intracerebral simulation and 1% in the intranasal simulation. Thus for cancer sites that are located further back in the brain away from the white matter tract, intranasal injection may not be a feasible option.

**Figure 10.**
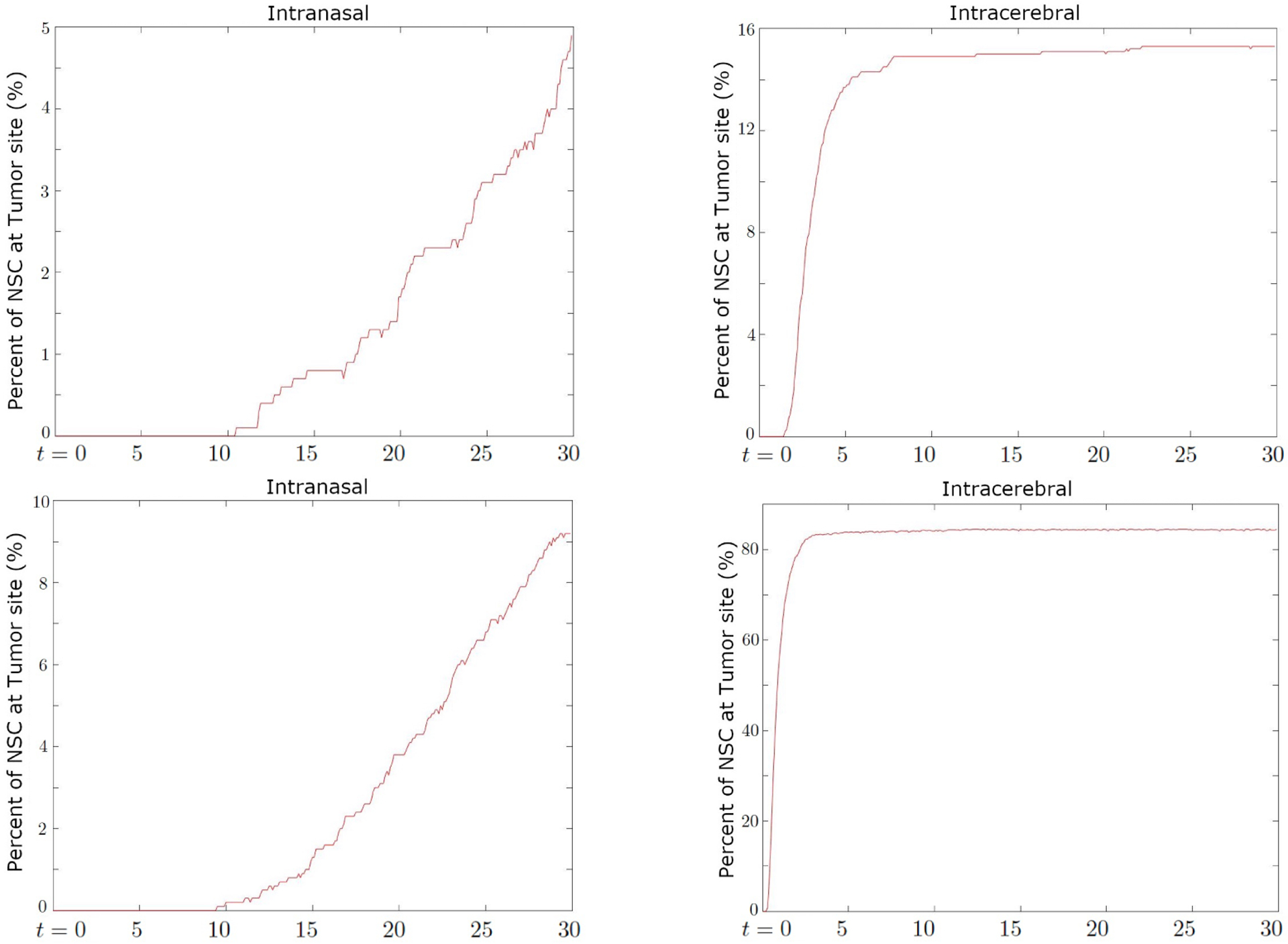
Case 3: Percentage of NSC that arrive at the cancer site on the left putamen (top) and at the cancer site on the right putamen (bottom) over time (days). The starting points are the intranasal injection site (left) and the intracerebral injection site (right). We can observe that more NSC reaches both cancer sites when injected intracerebrally, which is likely because this injection site is overall closer the cancer sites and begins on white matter. However, the distribution of NSCs among the two cancer site is more uniform when intranasally injected.

The third simulation has two cancer sites present, one centered at the front side of the right putamen, ***x***_*c*_ = [230, 300, 175] and the other centered at the middle of the left putamen ***x***_*c*_ = [400, 360, 155]. We observe that in intranasal injection, the NSC that took the shortest path to reach either cancer travels directly through the olfactory bulb to the cancer sites. This may be due to the closer location of the tumor from the injection site that some of the NSC can migrate with chemotactic driving force. On the other hand, the intracerebrally injected NSC has the advantage of being much closer to the cancer site on the right putamen, while the cancer site on the left putamen can be accessed through crossing the white matter tract to the other side of the brain. The direct result of this advantage can be seen in Figure 10, where the percentage of NSCs injected intracerebrally which reach the right cancer site is over 80%, and approximately 15% arrive at the left cancer site within two weeks. The proximity of the right cancer site to the injection causes the cells to stick to this site. This drawback has been observed in [6] from the experiment considering intracerebral/ventricular NSC administration to PBT017 glioma bearing mice. 1,640 NSC clusters arrived at the tumor on the same side of the injection, but only 230 clusters at the tumor on the opposite side. However, intranasal injection yields more evenly distributed NSCs from our simulation although the overall percentage is lower. Around 5% of NSCs arrive at the right cancer site and 9% arrive at the left cancer site. Considering the fact that intranasal injection can be given repeatedly, intranasal injection may yield a more uniform distribution of NSCs especially if there are multiple cancer sites at distinct locations.

## 4. Conclusions

In this paper, we develop an agent based model of therapeutic neural stem cell migration in a three-dimensional mouse brain with and without glioma brain tumor. As an extension to the original two-dimensional model in Rockne et al. (2018), our model allows us to examine the neural stem cell migration in a three-dimensional brain that elevates the potential usage of in silico simulation. In addition, the effect of uPA is added to model the chemotactic behavior of the neural stem cells, which makes them a promising therapeutic agent for cancer treatment. Finally, the stochasticity regarding the migration speed and sensitivity to chemotaxis is modeled with stochastic parameters. The effect of these added parameters on the migration distance, percentage in white matter tracts, and arrival percentage at the cancer sites are studied.

Using our model, we examine the efficacy of NSC treatment for different cancer locations and different injection strategies. In particular, we focus on comparing intranasal and intracerebral injections. Intranasal drug delivery provides an alternative and effective strategy to intracerebral injection which is more invasive. We compare the migration pattern of neural stem cells and tumor arrival rates of the two injection strategies. Considering the fraction of NSCs to arrive among injected NSCs, intracerebral injection is more effective due to its closer distance to the cancer site and injection location being on the white matter tract. However, due to such strong dependency on the injection location, when multiple cancer sites exist, the NSCs are concentrated at the nearest site which makes the distribution of NSCs less uniform compared to the intranasal injection. Although intranasal injection show a smaller arrival rate compared to intracerebral injection, NSCs are still able to follow the white matter tract all across the brain and a considerable amount of NSCs reach the cancer site. Moreover, when two tumor sites are located on opposite sides of the brain, intranasal injection yields a more even distribution compared to intracerebral injection. Considering that repeated administration is possible in intranasal delivery, our simulations supports the efficacy of intranasal delivery of NSC treatment, especially when there are multiple tumor sites across the brain.

Future work includes calibrating the model to experimental data and conducting parameter sensitivity analysis more carefully. We also plan to build on our model inspired by various cell migration modeling approaches and improve upon it. As discussed in Section 2.1, stochasticity can be added to the directed migration in our model. More sophisticated stochastic processes can be used, for instance cell position and velocity can be modeled as jump processes [57], where this approach enables incorporation of biochemical pathways into random walk description [58, 59]. On a similar note, modeling cell signaling networks can incorporate the feedback interaction between NSCs and chemoattractants [51]. In addition to the chemical component of the migration process, integrating the mechanical component is another possibility [60]. For instance, [61] derives and compares individual based mesoscopic model and population based macroscopic model to describe mesenchymal migration of cells in fibre networks, which provides us methods to model the NSC migration along the white matter fibers in multiple scales [62]. We also note that the model can be improved by including cell-cell interaction and contact inhibition [63]. Interaction between NSCs and glioma cells is another component to be added, in addition, to the interaction between NSCs, extracellular matrix, various cytokines and chemokines, and immune cells [13, 14, 64]. Phenomena of leader and follower cells [63, 65] in collective cell migration [66, 67] have been reported in many other systems including neural crest cells [68, 69] and we hope to investigate the possibility of such phenomena in NSC migration as well. In the future, the calibrated model can be used to study various dosing strategies. In particular, how to determine the dosage levels and how they should be spread out is an important question to be studied.

## Figures and Tables

**Table 1.** Model parameters, their biological interpretation, and their range of values. Values and ranges of *d*_*w*_ and *d*_*g*_ are taken from [10], and other parameters are estimated.

	biological meaning			
*M*_*coh*_(***x***)	Degree of antisotropy at ***x*** = (*x*, *y*, *z*)			
***M***_*ev*_(***x***)	Elongated direction of white matter at ***x***			
*C*(***x***)	Chemokine concentration at ***x***			
	biological meaning	value	units	reference
*d*_*w*_	Maximum NSC migration speed on white matter	[0.5, 5]	*μ*m day^−1^*	[10, 50]
*d*_*g*_	Maximum NSC migration rate on gray matter	[0.1, 5]	*μ*m day^−1^*	[10, 50]
*λ*_*c*_	Sensitivity to chemoattractant	[3, 10]	*μ*m day^−1^*	[50, 51]
*β*_*d*_	Stochasticity in migration speed	[1, 4]	1	[52, 53]
*α*_*c*_	Stochasticity in sensitivity to chemoattractant	[1, 5]	1	[51–53]
*ϵ*_*M*_	threshold of fractional anisotropy for white matter	0.4	1	[54]

* Additional scaling of multiplying with *X*/*T* is needed to convert the values to the written units, where X = 1.444 *μ*m for 2D DTI, X=13.5 *μ*m for 3D DTI, and *T* = Δ*t* = 1/1,000 *day*.

**Table 2.** Experimental results of HB1.F3.CD21 NSCs administered via intracerebral/ventricular [6] and intranasal [55] injection.

injection	glioma	time	dosage	arrival	reference
Intracerebral/ventricular	GL261	2 days	4 × 10^5^ cells	1321 ± 775.7 clusters	[6]
	PBT017	3 days	4 × 10^5^ cells	2076 clusters	
Intranasal	U251	1 day	6 × 10^5^ cells	3300 ± 934 cells	[55]
		4 days	6 × 10^5^ cells	7420 cells	

## Data Availability

All data and code used for running simulations and plotting is available on a GitHub repository at url: https://github.com/IllumiNate411/Mathematical-Modeling-of-Therapeutic-Neural-Stem-Cell-Migration-Towards-Brain-Tumors.
